# Blockade of CD155 and CD276 by Monoclonal Antibodies Fosters Immune Tolerance and Promotes Stable Engraftment of iPSC-Derived Islets in Allogeneic Humanized Mice

**DOI:** 10.3389/ti.2025.15433

**Published:** 2025-12-01

**Authors:** G. Siracusano, F. Deambrogio, V. Sordi, M. Malnati, L. Piemonti, R. Chimienti

**Affiliations:** 1 Diabetes Research Institute (DRI), IRCCS San Raffaele Scientific Institute, Milan, Italy; 2 University Vita-Salute San Raffaele, Milan, Italy; 3 Division of Immunology, Transplantation and Infectious Disease (DITID), IRCCS San Raffaele Scientific Institute, Milan, Italy

**Keywords:** monoclonal antibodies, immune tolerance induction, type 1 diabetes, allograft rejection, iPSC pancreatic derivatives

## Abstract

Induced pluripotent stem cell (iPSC)-derived pancreatic islets represent a promising therapeutic approach for restoring insulin independence in type 1 diabetes (T1D). However, their clinical success remains critically dependent on overcoming rejection mediated by innate and adaptive immune responses. Current immunosuppressive therapies pose significant long-term risks and only partially control alloimmune and autoimmune reactions. Targeted immunomodulation using monoclonal antibodies is a safer, more precise alternative. Here, we explored the impacts of blocking CD276 (B7-H3) and CD155 (PVR), activating ligands involved in immune recognition and regulation, on the survival and *in vivo* maturation of iPSC-derived endocrine progenitors (EPs) into functional pancreatic islets. Using a humanized mouse model, we demonstrated that dual blockade of CD276 and CD155 markedly reduced NK cell-mediated graft rejection, prevented CD14^+^ monocyte activation, and limited overall immune infiltration. In addition, CD155 blockade increased PD-1 levels on activated CD8^+^ T cells and significantly enhanced regulatory T cell (Treg) expansion and function, thereby promoting graft tolerance. Combined treatment prolonged engraftment and facilitated the maturation of EPs into functional, insulin-secreting cells, as indicated by increased human C-peptide levels and glucose responsiveness 4 weeks post-transplantation. Our findings highlight CD276/CD155 blockade as a novel immunomodulatory strategy to support tolerance and the functional maturation of iPSC-derived pancreatic grafts in T1D.

## Graphical Abstract

**Figure:**
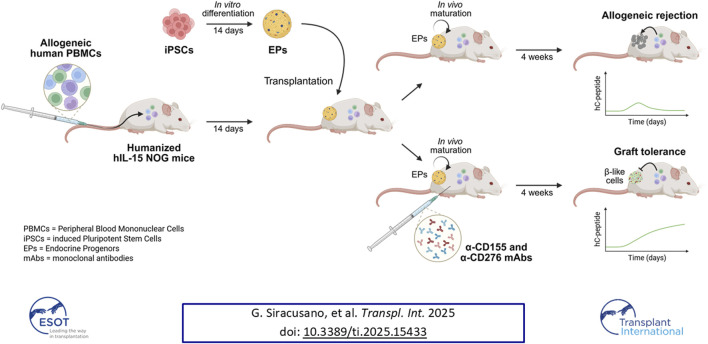

## Introduction

Induced pluripotent stem cell (iPSC) technology has ushered in a new era of regenerative medicine for treating type 1 diabetes (T1D), providing a potentially unlimited source of insulin-producing cells for transplantation and aiming to restore insulin independence [1–4]. Despite recent progress, the success of iPSC-derived β-cell transplantation still hinges on controlling complex immune responses [5, 6], requiring chronic immunosuppression to prevent rejection [7, 8]. However, standard regimens carry substantial long-term risks, including infections, malignancies, and metabolic complications [9], and they do not fully address the combined alloimmune and recurrent autoimmune responses typical of T1D recipients [10].

Innovative strategies have thus emerged to minimize or avoid chronic immunosuppression [11, 12]. These include genetically engineering stealthy, hypoimmunogenic stem cell-derived islets - which hold significant promise despite regulatory and safety challenges - and complementary approaches, such as the adoptive transfer of immunomodulatory cells or targeted induction therapies. Together, these methods aim to promote immune homeostasis and durable transplant tolerance by shifting from broad immunosuppression toward more precise immunomodulation.

Within this evolving landscape, monoclonal antibody (mAb) therapies that target specific immune pathways have long been considered an attractive strategy for preventing allograft rejection. For example, antibodies antagonizing the CD40/CD154 axis have been proposed to modulate alloimmune responses in preclinical animal transplant models [13, 14]. Although the early clinical development of anti-CD154 mAbs was halted after thromboembolic complications were reported in human studies [15], preclinical testing of anti-CD40 mAbs in rhesus macaques significantly reduced donor-specific antibody formation and prolonged islet survival [16]. Furthermore, anti-CD25 (basiliximab) is already used for induction therapy in islet transplantation for T1D patients [17, 18]. In renal allotransplantation, additional mAbs, such as anti-CD3 (OKT3) and anti-CD52 (alemtuzumab), have also reached clinical use, although with limited success [19, 20].

We previously demonstrated that genetically targeting the activating ligands CD276 (B7-H3) and CD155 (PVR) effectively prevents the recognition of MHC class I-deficient pancreatic cells by Natural Killer (NK) cells [21]. However, NK cells also contribute to allograft rejection through missing-self recognition in the context of Killer-cell Immunoglobulin-like Receptor (KIR)-HLA mismatches, where insufficient inhibitory signals due to mismatches promote NK-mediated graft loss [22, 23]. Activating ligands, such as CD276 and CD155, are crucial not only in direct NK cell recognition [21, 24, 25] but also in broader immune regulation [26–28]. These ligands play essential roles in modulating dendritic cell maturation [29, 30], macrophage-mediated recruitment and engulfment [31, 32], and lymphocyte T-cell activity [26, 33, 34], and thereby potentially impacting the entire immune response network [35]. In this study, we used monoclonal antibodies against CD276 and CD155, taking advantage of their prior preclinical and clinical development in oncology [36–40]. We assessed whether short-term peri-transplant administration of these targeted antibodies could foster immune tolerance by modulating key activating pathways, thereby improving engraftment and supporting the maturation of iPSC-derived pancreatic progenitors into functional islets.

## Materials and Methods

### Peripheral Blood Mononuclear Cell Isolation, iPSC Line Generation, and Differentiation

Peripheral blood mononuclear cells (PBMCs) for mouse humanization and iPSC generation were isolated from healthy donors after informed consent using Ficoll-Paque separation. Four iPSC lines (AMF70.10, NL83.01, RP84.03, and AG89.04) were generated by reprogramming the donor-derived PBMCs with the CytoTune-iPS 2.0 Sendai Reprogramming Kit (Thermo Fisher Scientific). All lines were routinely screened for *Mycoplasma* using the MycoAlert Detection Kit (Lonza). Luciferase gene transduction was performed as previously described [21], and pancreatic differentiation followed established protocols [41].

### NK Cell Isolation and Expansion

NK cells were isolated from freshly obtained donor PBMCs using the CD56^+^CD16^+^ NK Cell Isolation Kit (Miltenyi Biotec) and expanded for 12 days in NK MACS Medium (Miltenyi Biotec) supplemented with 5% human AB serum (Corning), 70 ng/mL IL-15, and 500 U/mL IL-2 (Peprotech).

### KIR-HLA Interaction Scoring

We quantified donor NK cell-recipient iPSC compatibility using an additive score that integrates the inhibitory (L) and activating (S) KIR copy numbers with the recipient HLA-C group and Bw4 epitopes. Copy numbers were obtained from KIR genotyping (per gene), while the HLA-C group and Bw4-I80/T80 were derived from iPSC HLA typing. A positive total score (S > 0.0) indicates a net inhibitory match, a negative score (S < 0.0) indicates a net activating/missing-self-like mismatch, and a score of zero (S = 0.0) is neutral. The Bw4 weight was defined as:

$$W_{\text{BW}4}={1.5n}_{I80}+{1.0n}_{T80}$$

For C1/C1 recipients:

$$S_{C1/C1}=+{1K}_{2\text{DL}2}n_{C1}+0.5_{2\text{DL}3}n_{C1}-{1K}_{2\text{DL}1}n_{C1}-{1K}_{2\text{DS}2}n_{C1}+{1K}_{2\text{DS}1}+n_{C1}+{0.5K}_{2\text{DS}5}n_{C1}+{1K}_{3\text{DL}1}w_{\text{Bw}4}-{1K}_{3\text{DS}1}w_{\text{Bw}4}-{0.1K}_{2\text{DS}4f}n_{C*04:01}-{0.05K}_{2\text{DS}4f}n_{A11}+{0.1K}_{3\text{DL}2}n_{A11}$$

For C2/C2 recipients:

$$S_{C2/C2}=+{1K}_{2\text{DL}1}n_{C2}-{0.6K}_{2\text{DL}2}n_{C2}-{0.1K}_{2\text{DL}3}n_{C2}-{1K}_{2\text{DS}1}n_{C2}+{1K}_{2\text{DS}2}n_{C2}-{0.5K}_{2\text{DS}5}n_{C2}+{1K}_{3\text{DL}1}w_{\text{Bw}4}-{1K}_{3\text{DS}1}w_{\text{Bw}4}-{0.1K}_{2\text{DS}4f}n_{C*04:01}-{0.05K}_{2\text{DS}4f}n_{A11}+{0.1K}_{3\text{DL}2}n_{A11}$$

Where “K” represents the copy number of the given KIR gene, and “n” represents the iPSC allele counts for the corresponding HLA determinant. Interaction weights were based on published KIR-HLA interactions and included a dedicated term for KIR2DS4-HLA-C*04:01 binding, along with additional terms for KIR2DS4 and KIR3DL2 in the presence of HLA-A*11:01.

### *In Vitro* Cytotoxicity Assays

Endocrine progenitor (EP) clusters were cultured in suspension at 95 rpm in their specific medium, which was supplemented with 10 ng/mL IFN-γ and 50 ng/mL TNF-α (Peprotech). NK cells were pre-activated with 1,000 U/mL IL-2 and 20 ng/mL IL-12 (Peprotech). After overnight incubation, the iPSCs or the EP clusters were stained with 250 nM Incucyte Cytotox Green Dye (Sartorius) in complete NK MACS medium. The NK cell effectors were labeled with 5 µM Cell Proliferation Dye eFluor670 (Thermo Fisher Scientific). The effector and target cells were co-cultured at a 1:1 ratio in 96-well plates, with ≥3 target-only wells included to quantify basal cell death. The plates were then placed in the IncuCyte S3 Live-Cell Analysis System and analyzed using the associated software (Sartorius).

### *In Vivo* Experiments

Female NOD-scid IL2Rgammanull (NSG) mice (age: 6–8 weeks old; weight: 20–24 g) were obtained from Charles River Laboratories, Italy. Female hIL-15 NOG mice (NOD.Cg-*Prkdc* ^*scid*^ *Il2rg* ^*tm1Sug*^ Tg (CMV-IL2/IL15)1-1Jic/JicTac) (age: 6–8 weeks old; weight: 20–24 g) were purchased from Taconic Biosciences. Female mice were chosen because males more often develop spontaneous dermatitis, which could confound symptoms of graft-versus-host disease (GvHD). Additionally, female mice exhibit more consistent human immune cell engraftment. The mice were humanized via an intravenous injection of 2.5 × 10^6^ human PBMCs, with PBMC labeling using the IVISense DiR 750 Fluorescent Cell Labeling Dye (XenoLight, Revvity, Inc.) performed as needed, following the manufacturer’s instructions. After 14 days, the mice were infused with ∼800 clusters (100–120 μm in diameter) into the intermuscular space of their lower hindlimbs and were monitored for up to 4 weeks post-transplantation. All procedures were conducted under protocols approved and overseen by the Animal Care and Use Committee of the San Raffaele Scientific Institute.

### *In Vivo* Imaging

D-luciferin potassium salt (PerkinElmer) was administered intraperitoneally to anesthetized mice at 150 mg/kg. The animals were imaged using the Lumina II IVIS system (PerkinElmer), acquiring both bioluminescence and fluorescence signals. Bioluminescence was quantified as maximum radiance expressed in photons/s/cm^2^/sr, whereas fluorescence was measured as average radiant efficiency ([photons/s/cm^2^/sr]/[µW/cm^2^]). Signal intensities within the defined Regions of Interest (ROIs) were quantified using Aura software (Spectral Instruments Imaging).

### Flow Cytometry

Clusters were dissociated with trypsin (Lonza), and live cells were identified using the LIVE/DEAD Fixable Violet kit (Thermo Fisher Scientific). Surface staining was performed by incubating cells with antibodies for 30 min at 4 °C in FACS buffer (DPBS with 2% FBS and 2 mM EDTA). For intracellular staining, cells were fixed with Cytofix/Cytoperm (BD Biosciences) and permeabilized with Phosflow Perm Buffer III (BD Biosciences), then incubated for 45 min at 4 °C with intracellular antibodies. For the phenotyping of humanized mice, 50 µL of blood collected from the retro-orbital plexus was stained for 30 min at 4 °C, followed by red blood cell lysis with BD FACS™ Lysing Solution prior to analysis. The following conjugated antibodies were used: anti-B2M-APC (clone 2M2); anti- HLA-A, B, C-PE (clone W6/32), anti-CD45-PE/Dazzle™594 (clone HI30); anti-CD8-BV605 (clone SK1); anti-CD154-APC (clone 24–31) (all were obtained from Biolegend); anti-OCT3/4-AF647 (clone 40/Oct3); anti-CD184-PE (clone 12G5); anti-PDX-1-AF488 (clone 658A5); anti-NKX6.1-PE (clone R11-560); anti-insulin-AF647 (clone T56-706); anti-glucagon-BV421; anti-CD3-FITC/BUV395 (clone SK7); anti-CD4-PB (clone RP-T4); anti-CD56-PE (clone NCAM1); anti-HLA-DR-BV480 (clone G46-6); a-PD-1-R718 (clone EH12.1); anti-CD38-APC (clone HIT2); anti-CD223-BUV395 (clone T47-530), anti-FoxP3-R718 (clone 259D/C7); anti-Helios-PE (clone 22F6); anti-CD107a-APC-H7 (clone H4A3) (all were obtained from BD Biosciences); anti-CD4-PE-Vio770 (clone REA623); anti-CD14-VioGreen (clone TÜK4) (both were obtained from Miltenyi Biotec); anti-CD16-SuperBright436 (clone 3G8) (obtained from Invitrogen); and anti-CD69-StarBright UltraViolet 510 (clone FN50) (obtained from BioRad). The cells were acquired on the CytoFLEX LX flow cytometer (Beckman Coulter) using CytExpert, and the data were analyzed using FlowJo v10.

### Glucose Tolerance Test and C-Peptide Measurement

An intraperitoneal glucose tolerance test (ipGTT) was performed on day 28. After a 4-h fast, the mice received a 2 g/kg intraperitoneal glucose bolus, and blood glucose was monitored at 0, 30, 60, 90, and 120 min. At the 90-min time point, blood was collected and plasma was isolated by centrifugation and subsequently analyzed for human C-peptide using a Mercodia ELISA. Absorbance was measured on a BioRad microplate reader.

### Immunohistochemistry

Explanted grafts were fixed, processed, and paraffin-embedded. Sections were cut at 5 µm for histological analysis. Hematoxylin and eosin (H&E) staining was performed to visualize the grafts and assess their morphology. BOND™ Ready-To-Use Primary Antibody Insulin (Leica, clone 2D11-H5) was used to detect mature iPSC-derived islets. The slides were scanned using a Leica Aperio 200.

### Statistical Analysis

All data are presented as the mean ± SEM unless otherwise specified. Comparisons between more than two independent groups were performed using one-way ANOVA with a Tukey’s *post hoc* test or a Kruskal-Wallis with Dunn’s test for non-parametric data. Longitudinal datasets were analyzed using two-way ANOVA followed by Holm-Šídák’s correction. Survival analysis was conducted using the Kaplan-Meier method with the log-rank (Mantel-Cox) test. Pairwise comparisons were made using a two-tailed unpaired or paired Student’s t-test, as appropriate. All analyses were performed using GraphPad Prism v10.

## Results

### Blockade of CD276 and CD155 Dampens Missing-Self-Recognition and Killing of SC-Derived Pancreatic Endocrine Cells by KIR-HLA Mismatched NK Cells

**FIGURE 1:**
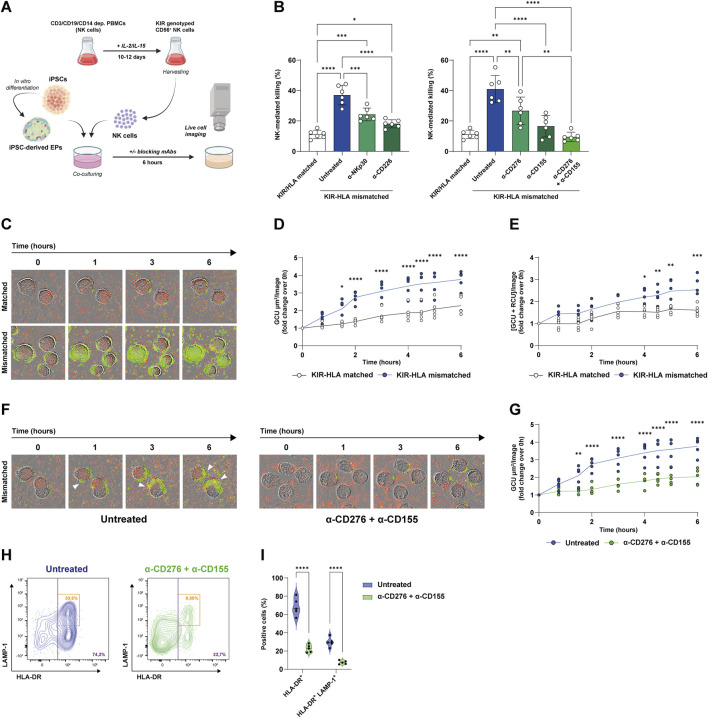
Blockade of CD276 and CD155 reduces missing-self recognition and NK-mediated killing of iPSC-derived EPs. **(A)** Experimental workflow. Both iPSCs and iPSC-derived EP clusters were co-cultured with KIR-genotyped NK cells that were isolated and expanded from healthy donor PBMCs in the presence of IL-2 and IL-15. NK-mediated cytotoxicity was evaluated by live-cell imaging over 6 h, in the presence or absence of blocking mAbs targeting the NKp30/CD276 and CD226/CD155 axes. **(B)** Quantification of NK-mediated killing (% of dead target cells) under KIR-HLA-matched (white bars) or mismatched (colored bars) conditions, with the latter in the presence of α-NKp30, α-CD226, α-CD276, α-CD155, or both α-CD276 and α-CD155 mAbs. N = 6. *p < 0.05; **p < 0.01, ***p < 0.001, ****p < 0.0001. **(C)** Representative time-lapse images of NK cell infiltration and EP cluster lysis under matched and mismatched conditions (green = dead cells, red = eFluor670-labeled human NK cells). **(D)** Kinetic analysis of dead cell area expressed as Green Calibrated Unit (GCU) per square micrometer per field of view, under matched versus mismatched conditions. The line represents the mean. N = 5. *p < 0.05, ****p < 0.0001 by two-way ANOVA followed by Šídák’s *post hoc* multiple comparison test. **(E)** Kinetic analysis of NK cell-mediated infiltration and killing measured as the co-localization of GCU and Red Calibrated Unit (RCU) per field of view, under matched versus mismatched conditions. The line represents the mean N = 5. *p < 0.05, **p < 0.01, ***p < 0.001 by two-way ANOVA followed by Šídák’s *post hoc* multiple comparison test. **(F)** Representative time-lapse images of NK cell infiltration and killing in untreated versus α-CD276 + α-CD155-treated EPs under mismatched conditions. **(G)** Kinetic analysis of dead cell area under untreated versus α-CD276 + α-CD155-treated EPs. The line represents the mean. N = 5. **p < 0.01, ****p < 0.0001 by two-way ANOVA followed by Šídák’s *post hoc* multiple comparison test. **(H)** Flow cytometry plots showing NK cell activation (HLA-DR^+^) and degranulation (LAMP-1^+^) under untreated versus α-CD276 + α-CD155 treated conditions. **(I)** Violin plots representing the quantification of HLA-DR^+^ and HLA-DR^+^/LAMP-1^+^ NK cells. N = 5. ****p < 0.0001 by two-tailed unpaired Student’s t-test.;

First, we examined the contribution of the NKp30-CD276 and CD226-CD155 axes to missing-self recognition under KIR-HLA class I mismatch conditions using *in vitro* NK cytotoxicity assays. The NK cells derived from eight KIR-genotyped donors were matched or mismatched with four HLA-typed iPSC lines. Undifferentiated iPSC lines were then co-cultured with the NK cells, and NK-mediated killing events were recorded via live cell microscopy in the presence or absence of blocking mAbs (Figure 1A). The list of characteristics of both donor NK cells and iPSC lines used in these experiments, along with the matched/mismatched pairs based on KIR gene copy number and HLA haplotype, is reported in Supplementary Tables S1-S3.

As expected, KIR-HLA mismatching resulted in a significant three-to-fourfold increase in NK-mediated killing compared to matched pairs (11.1% ± 2.7% vs. 36.9% ± 6.4%; p < 0.0001). Blocking either NKp30 or CD226 with specific mAbs reduced the killing of mismatched cells (24.2% ± 4.2% with α-NKp and 18.1% ± 2.6% with α-CD226, respectively) (Figure 1B). Accordingly, blocking the activating ligands CD276 or CD155 on target cells reduced NK cell cytotoxicity by a similar amount as blocking their counterreceptors (26.7% ± 8.9% with α-CD276 and 16.5% ± 7.0% with α-CD155, respectively). Remarkably, the combination of CD276 and CD155 blockade had a synergistic effect, reducing NK-mediated killing of the mismatched iPSCs to levels comparable to those of KIR-HLA-matched pairs (11.1% ± 2.7% for matched vs. 40.9% ± 8.9% for mismatched vs. 9.6% ± 3.0% with α-CD276+α-CD155, respectively) (Figure 1B).

Previously, we demonstrated that both β2-microglobulin (B2M) and HLA class I molecules are dynamically regulated during pancreatic differentiation [21]. Consistent with prior findings, iPSC lines exhibited the highest B2M and HLA-A/B/C expression levels, while a marked downregulation was observed at the posterior foregut (PF) and EP stages. SC-islets displayed an intermediate and more heterogeneous expression profile, with a broader range of HLA class I surface levels among cells (Supplementary Figures S1A,B).

Since EP clusters show low HLA expression, potentially limiting T cell immunogenicity, we hypothesized that they might be susceptible to missing-self-recognition by NK cells. Supplementary Figures S1C–E illustrate changes in key differentiation markers at each stage. OCT4 was highly expressed in iPSCs and was rapidly lost after definitive endoderm (DE) induction, while CXCR4 and FOXA2 increased. Progression to the EP stage was associated with increased expression of the pancreatic lineage markers PDX1 and NKX6.1, along with the early endocrine markers INS and GCG (Supplementary Figure S1C). Coexpression of PDX1 and NKX6.1 in EP cells was confirmed by flow cytometry (Supplementary Figure S1D), and EP cluster morphology was documented by bright-field imaging (Supplementary Figure S1E). To evaluate the susceptibility of EP clusters to NK cell infiltration and killing *in vitro*, we performed live-cell imaging cytotoxicity assays under both KIR-HLA matching and mismatching conditions. In mismatched pairs, missing-self-recognition occurred, as evidenced by increased NK cell infiltration and EP cell killing. This was measured by a progressive rise in green fluorescence over time (Figures 1C,D) and increased co-localization of red-labeled NK cells with green fluorescence signals (Figure 1E). Importantly, treatment of HLA-mismatched EPs with α-CD276 and α-CD155 monoclonal antibodies significantly reduced cell death (Figures 1F,G), in addition to NK cell activation markers, including HLA-DR (68.4% ± 9.7% vs. 22.7% ± 4.2%; p < 0.0001) and the degranulation marker CD107a (LAMP-1) on activated HLA-DR^+^ cells (29.7% ± 5.1% vs. 7.5% ± 1.9%; p < 0.0001) (Figures 1H,I).

### Dual Blockade of CD276 and CD155 Prevents Immune Rejection of EP Grafts in a Fully Humanized Mouse Model

**FIGURE 2:**
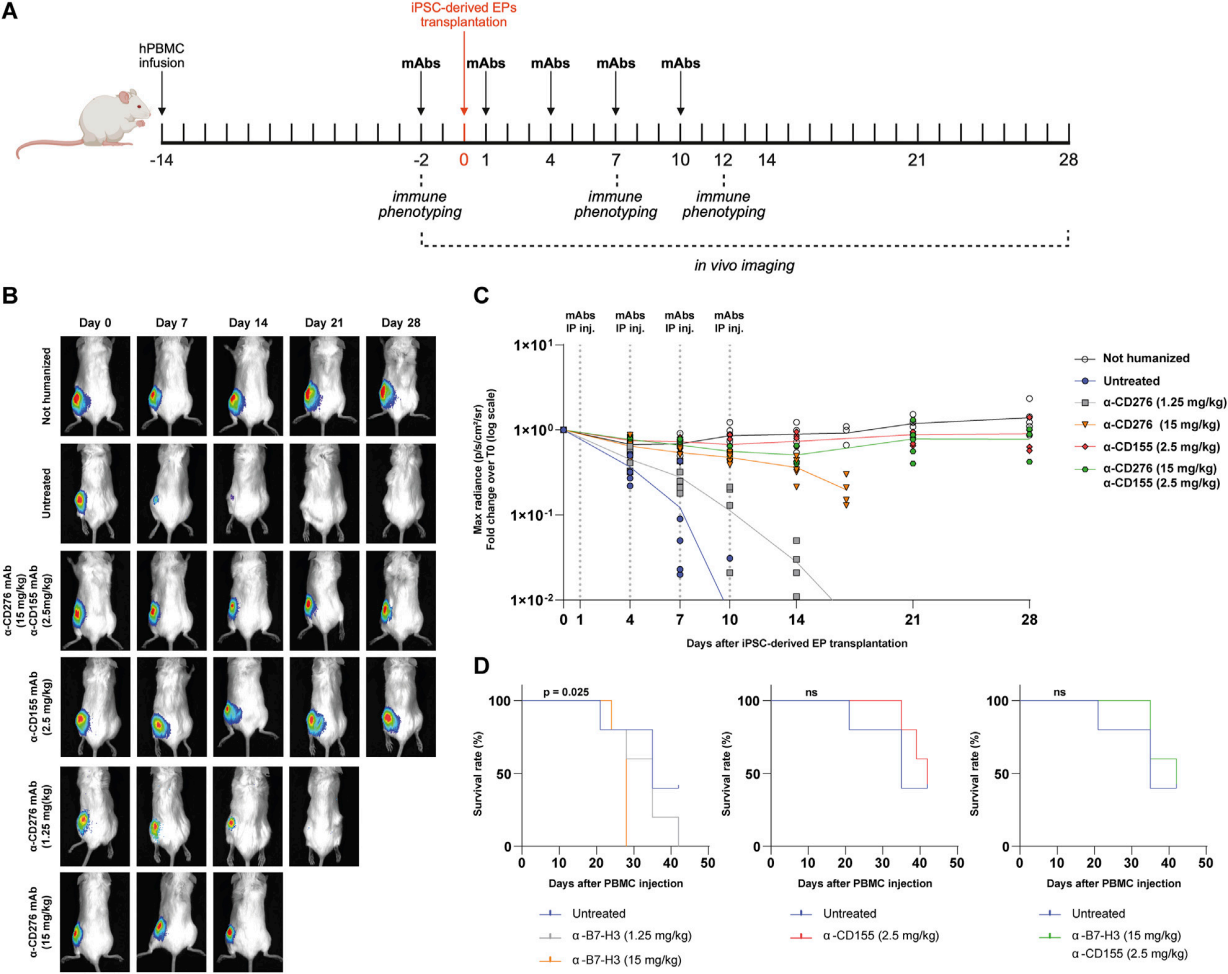
*In vivo* blockade of CD276 and CD155 enhances the survival of iPSC-derived EP grafts in a humanized mouse model. **(A)** Schematic overview of the experimental design for the *in vivo* evaluation of allograft rejection of iPSC-derived EPs in humanized hIL-15 NOG mice. **(B)** Representative bioluminescence imaging (BLI) of transplanted EP clusters at different time points (day 0 to day 28) in untreated mice or mice receiving α-CD276 and/or α-CD155 mAbs at the indicated doses. **(C)** Quantification of the BLI signal (max radiance, p/s/cm^2^/sr) over time, normalized to the signal on day 0. N = 5 mice per group. Statistical significance was determined by two-way ANOVA followed by Šídák’s *post hoc* multiple comparison test. **(D)** Kaplan-Meier survival curves of humanized mice under different treatment conditions (untreated, α-B7-H3, α-CD155, or combined therapy). Statistical significance was assessed using the log-rank (Mantel–Cox) test.

Since CD276 and CD155 are expressed on both graft cells and immune cells, we next evaluated whether antagonizing them may provide graft tolerance by modulating immune cell activity other than direct graft targeting. NSG mice demonstrated sustained engraftment of T lymphocytes but limited NK cell and monocyte reconstitution (Supplementary Figure S2); therefore, we used hIL-15 NOG immunodeficient mice, which achieve superior humanization efficiency and improved engraftment of innate immune cells (Supplementary Figure S3). To evaluate the efficacy of blocking mAbs, mice were first injected intravenously with 2.5 × 10^6^ human PBMCs and, 14 days later, transplanted with ∼800 clusters of luciferase-expressing iPSC-derived EPs into the intermuscular space of the lower hindlimbs. The mAb treatment regimen consisted of 5 intraperitoneal injections every 3 days, starting 2 days before transplantation and continuing up to 10 days post-transplantation, as outlined in Figure 2A. We tested two doses of α-CD276 (1.25 and 15 mg/kg), one dose of α-CD155 (2.5 mg/kg), and a combined treatment consisting of the highest dose of α-CD276 (15 mg/kg) plus α-CD155 (2.5 mg/kg). Humanized untreated mice rejected EP grafts within 7–10 days (Figures 2B,C). Notably, increasing doses of α-CD276 prolonged graft survival for 2 weeks (Figure 2C), but were associated with increased mortality due to an earlier onset of GvHD, as confirmed by log-rank trend analysis (p = 0.025) (Figure 2D). In contrast, treatment with α-CD155, either alone or in combination with α-CD276, ensured graft survival for up to 4 weeks at levels comparable to non-humanized mice (Figure 2C). Moreover, co-administration of α-CD155 mitigated the severe GvHD effects observed with α-CD276 alone, as mortality in the α-CD155-treated groups was comparable to that of untreated humanized mice (Figure 2D).

### *In Vivo* Imaging Reveals Reduced PBMC Accumulation at the Implant Site Under CD276/CD155 Blockade

**FIGURE 3:**
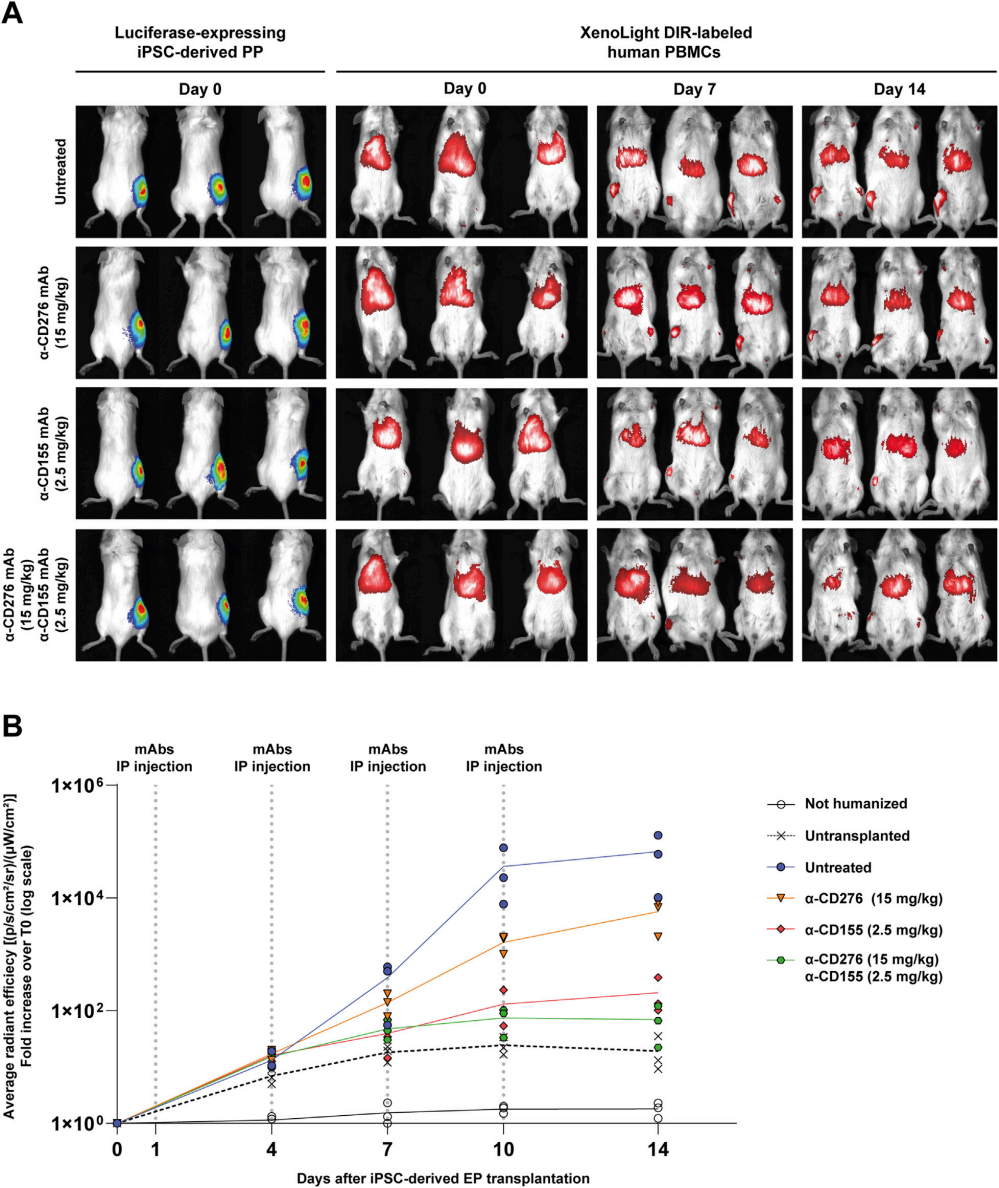
*In vivo* tracking of human PBMCs after iPSC-derived EP transplantation under CD276/CD155 blockade. **(A)** Representative bioluminescence imaging (BLI) of luciferase-expressing iPSC-derived EPs and XenoLight DiR-labeled human PBMCs in hIL-15 NOG mice. Images were acquired before transplantation (TX) and 7 and 14 days after under untreated or α-CD276, α-CD155, and combined mAb treatment conditions. **(B)** The average radiant efficiency [(p/s/cm^2^/sr)/μW/cm^2^)] of the graft area is expressed as a fold change compared to the signal recorded on day 0 to measure PBMC infiltration. Untransplanted humanized mice and non-humanized mice were used as controls. N = 3. Statistical significance between groups at each time point was assessed using a Kruskal-Wallis test followed by Dunn’s *post hoc* multiple comparison test.

The kinetics of immune cell infiltration and the impact of checkpoint blockade were longitudinally investigated by *in vivo* imaging combining bioluminescence (to track luciferase-expressing EP grafts) and fluorescence detection of DiR-labeled human PBMCs (Figure 3A). Humanized mice were treated intraperitoneally with α-CD276 (15 mg/kg), α-CD155 (2.5 mg/kg), or both antibodies in combination at the same doses.

As early as day 4, untreated humanized mice showed progressive PBMC accumulation at the graft site, with the fluorescence signal increasing from 13.2 ± 5.0- to 66,537 ± 59,820-fold over background by day 14. Mice treated with α-CD276 alone showed delayed yet substantial infiltration (5,727 ± 3,284 fold), while α-CD155 alone more effectively reduced immune cell recruitment (208 ± 158 fold). Strikingly, the combined blockade of CD276 and CD155 limited the increase in PBMC-associated fluorescence on day 14 to ∼69-fold over the day 0 baseline. Although this signal remains higher than that observed in untransplanted (∼19-fold) and non-humanized (∼1.8-fold) mice at the same time point, the absence of further fluorescence amplification indicates that only a few immune cells reach the grafts, without evidence of sustained recruitment (Figure 3B).

No significant differences were observed between groups at early time points (day 4–10). On day 14, however, Kruskal-Wallis analysis revealed significant divergence in PBMC accumulation across experimental groups (H = 11.48, p = 0.043). Dunn’s *post hoc* comparisons revealed that PBMC infiltration in untreated mice (66,537 ± 59,820-fold) was significantly higher than in non-humanized (1.81 ± 0.54-fold; p < 0.001), untransplanted (19.2 ± 14.0-fold; p < 0.01), and dual mAb-treated mice (69.6 ± 49.1-fold; p < 0.05). Differences with α-CD276 (5,727 ± 3,284-fold) and α-CD155 (208 ± 158-fold) were not statistically significant after correction, although a downward trend was noted in the α-CD155 group (Figure 3B).

### Anti-CD276 Prevents Innate Immune Cell Activation and Migration, While the CD155 Blockade Enhances T cell Exhaustion and Promotes CD4^+^ Treg Expansion

**FIGURE 4:**
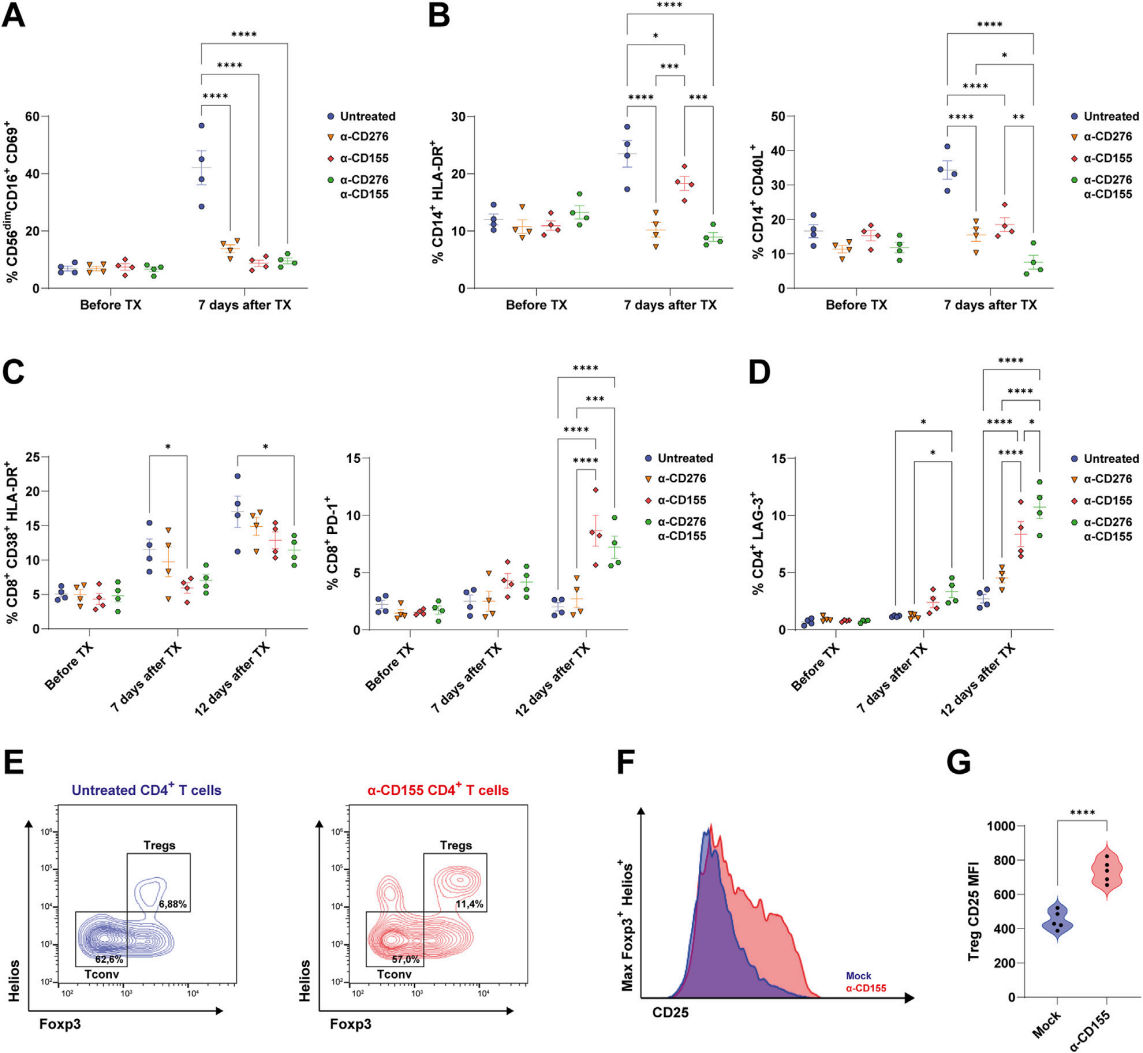
Blockade of CD276 and CD155 modulates the activation of human immune cells. **(A)** Frequency of activated NK cells (CD56^+^CD16^+^CD69^+^) in the peripheral blood before and 7 days after EP TX in untreated or antibody-treated mice (α-CD276, α-CD155, or combination therapy). Data represent mean ± SEM. N = 4. ****p < 0.0001 by one-way ANOVA followed by Tukey’s *post hoc* multiple comparison test. **(B)** Frequencies of activated monocytes, evaluated by expression of HLA-DR and CD40L, before and 7 days after TX. Data represent mean ± SEM. N = 4. *p < 0.05, **p < 0.01, ***p < 0.001, ****p < 0.0001 by one-way ANOVA followed by Tukey’s *post hoc* multiple comparison test. **(C)** Frequencies of activated CD8^+^ T cells (CD8^+^CD38^+^HLA-DR^+^) and PD-1^+^CD8^+^ T cells before, 7 days, and 12 days after TX. Data represent mean ± SEM. N = 4. *p < 0.05, ***p < 0.001, ****p < 0.0001 by one-way ANOVA followed by Tukey’s *post hoc* multiple comparison test. **(D)** Frequency of CD4^+^ T cells expressing LAG-3 before and 7 and 12 days after TX. Data represent mean ± SEM. N = 4. *p < 0.05, ***p < 0.001, ****p < 0.0001 by one-way ANOVA followed by Tukey’s *post hoc* multiple comparison test. **(E)** Representative flow cytometry plots of CD4^+^ T cells showing Tconv (Foxp3^−^Helios^-^) and Treg (Foxp3^+^Helios^+^) subsets under untreated and α-CD155-treated conditions. **(F)** CD25 expression on Foxp3^+^Helios^+^ Tregs under untreated and α-CD155-treated groups. **(G)** Violin plots showing the quantification of the CD25 Mean Fluorescent Intensity (MFI) on Foxp3^+^Helios^+^ Tregs. N = 5. ****p < 0.0001 by two-tailed unpaired Student’s t-test.

Next, we sought to investigate the mechanisms underlying the protective effects of α-CD276 and α-CD155 mAb treatments and their impact on immune cell activation and migration. According to *in vitro* experiments, we found that both α-CD276 and α-CD155 prevented the overexpression of the tissue migration marker CD69 on circulating CD56^dim^CD16^+^ NK cells (Figure 4A). Moreover, α-CD276, either alone or in combination with α-CD155, dampened CD14^+^ monocyte activation and migration, as confirmed by lower levels of HLA-DR^+^ and CD40L^+^ cells compared to the untreated group (Figure 4B), supporting the hypothesis that CD276 signaling contributes to monocyte infiltration into the grafts.

However, high-dose α-CD276 alone did not prevent CD8^+^ T cell activation (Figure 3C). In contrast, α-CD155 treatment alone significantly increased the frequency of exhausted PD-1^+^CD8^+^ T cells (Figure 4C) and LAG-3^+^CD4^+^ T cells (Figure 4D) 12 days post-transplantation. The combination of α-CD276 and α-CD155 led to a slight but significant reduction in CD38^+^HLA-DR^+^CD8^+^ T cells at 12 days post-transplantation (Figure 4C) and an early increase in LAG-3 expression on total CD4^+^ T cells compared to the untreated group (Figure 4D). In line with recent studies showing that CD226 signaling negatively affects Treg stability [42, 43], blockade of its ligand CD155 resulted in a twofold increase in the percentage of total Tregs compared to the untreated mice (Figure 4E). Furthermore, evaluation of IL-2 Receptor alpha (IL-2Rα/CD25) on FoxP3^+^Helios^+^CD4^+^ Tregs revealed a significant upregulation of CD25 surface expression in the α-CD155-treated group compared to mock controls (735 ± 66.4 vs. 488 ± 53.6; p < 0.0001) (Figures 4F,G), supporting the hypothesis of an enhanced Treg suppressive ability in response to CD155 blockade, consistent with previous reports [44, 45].

### EP Grafts Properly Mature Into Glucose-Responsive, Insulin-Secreting β Cells in mAb-Treated Humanized Mice

**FIGURE 5:**
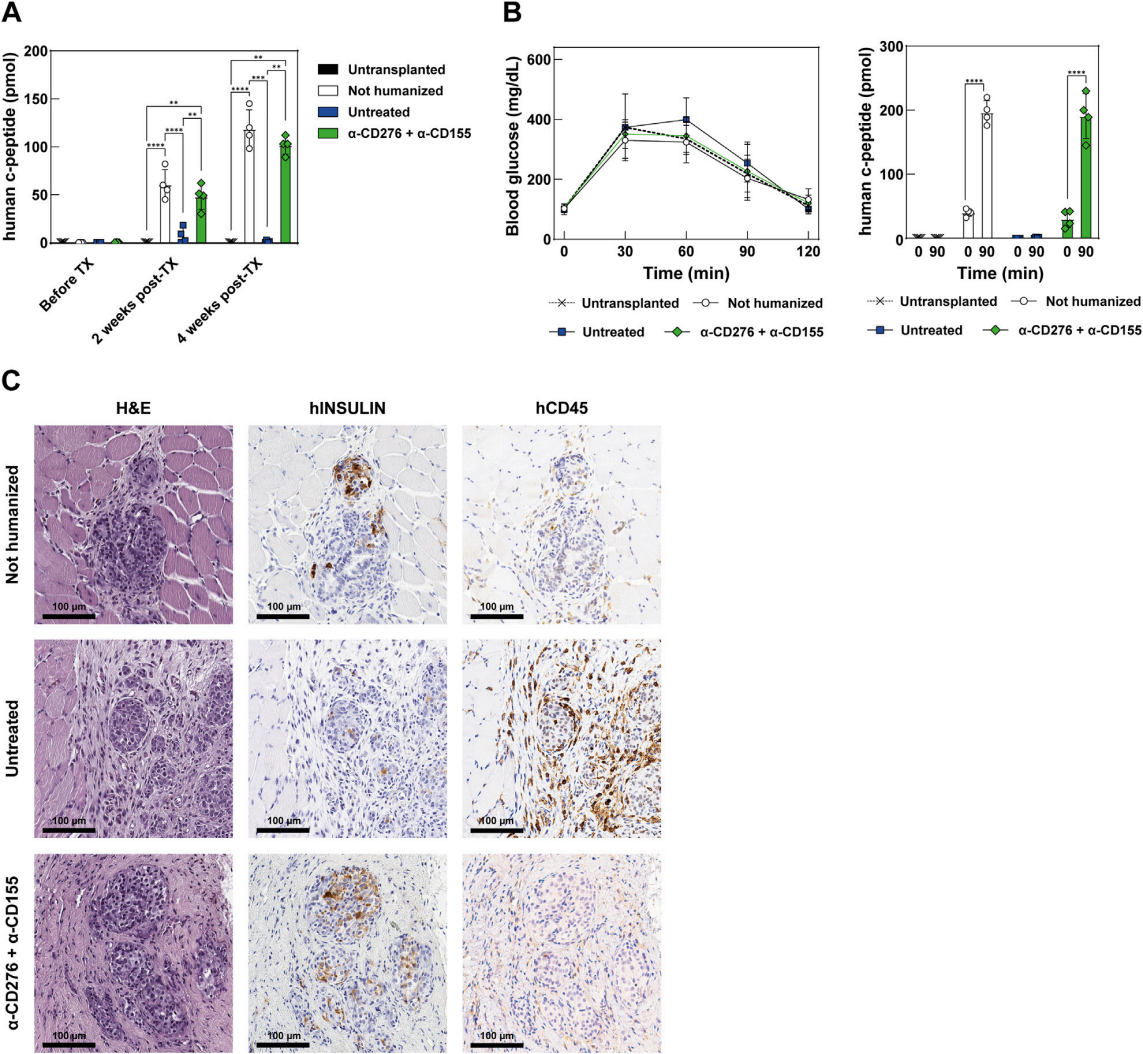
Functional assessment and maturation of EP grafts after transplantation **(A)** Quantification of circulating human C-peptide in the plasma of recipient mice at baseline (before TX), 2 weeks, and 4 weeks post-TX. Data represent mean ± SD. N = 4. **p < 0.01, ***p < 0.001, ****p < 0.0001 by one-way ANOVA followed by Tukey’s *post hoc* multiple comparison test. **(B)** Intraperitoneal glucose tolerance test (ipGTT) at 28 days post-TX. The left panel shows blood glucose concentrations in mg/dL over time; the right panel shows human C-peptide secretion at baseline and 90 min post-glucose challenge. Data represent mean ± SD. N = 4. ****p < 0.0001 by two-tailed paired Student’s t-test. **(C)** Representative histology of the graft site at 28 days post-TX in non-humanized, untreated, or treated mice receiving the combined α-CD276 and α-CD155 treatment. The H&E-stained section shows a pancreatic graft overlying host muscle fibers. Human insulin-positive endocrine cells and infiltrating CD45-positive immune cells are also visible. Scale bar: 100 μm.

To assess the maturation and function of the iPSC-derived EP grafts, we monitored plasma human c-peptide levels for 4 weeks post-transplantation. A progressive increase in basal c-peptide levels was observed in both non-humanized and mAb-treated mice, indicating functional maturation. In stark contrast, humanized mice that did not receive CD276/CD155 blockade showed only a modest increase in c-peptide levels at 2 weeks (7.27 ± 8.11 pmol), followed by a complete loss at 4 weeks, consistent with the early rejection of endocrine cells and the failure of the grafts to mature (Figure 5A).

On day 28, an ipGTT was performed to evaluate glucose responsiveness. It should be noted that the glycemic curves shown are not intended to reflect graft functionality, as glycemia is predominantly controlled by endogenous murine islets in this setting. Both non-humanized and mAb-treated mice exhibited a significant rise in human c-peptide 90 min after glucose administration (196.5 ± 18.6 vs. 40.0 ± 5.88 pmol and 191.0 ± 35.2 vs. 30.2 ± 13.4 pmol, respectively; p < 0.0001), while the untreated humanized group showed a negligible response at 90 min post-ipGTT (1.02 ± 0.79 pmol), indicating an absence of functional endocrine cells (Figure 5B).

Histological analysis showed preserved graft architecture in non-humanized and mAb-treated mice, whereas untreated mice displayed tissue disorganization. Insulin-producing cells were found in both non-humanized and mAb-treated groups, but not in the untreated group, consistent with the c-peptide data. Moreover, human CD45 staining showed marked leukocyte infiltration in the grafts of the untreated mice, but not in the mAb-treated groups (Figure 5C). These findings indicate that dual blockade of CD276 and CD155 protects the grafts from early immune rejection, enabling *in vivo* maturation of EP cells into functional, glucose-responsive β-like cells.

## Discussion

Treatment with α-CD276 and α-CD155 blocking monoclonal antibodies in humanized hIL-15 NOG mice transplanted with iPSC-derived EPs significantly prevented immune activation and allorejection. Our study provides the first preclinical proof-of-concept for targeting stress-inducible ligands such as CD276 (B7-H3) and CD155 (PVR), as a strategy to promote graft tolerance through selective immunomodulation. These findings expand on previous evidence showing that both ligands mediate NK cell-driven missing-self recognition [21, 46] and broadly shape immune crosstalk during inflammatory responses [47, 48].

CD276, a B7-family immune checkpoint, has been extensively investigated in oncology; however, its function remains debated due to its dual activating/inhibiting effects [49]. Originally identified as a co-stimulatory molecule that interacts with NKp30, CD276 can deliver both activating and inhibitory signals through the TLT-2 (TREML2) receptor [50, 51]. While the function of TLT-2 in NK cells remains uncertain [52], its expression on monocytes, macrophages, and granulocytes has been linked to enhanced phagocytic activity and IL-6 production [32, 53]. In transplantation, CD276 has been implicated in both acute and chronic islet allograft rejection [54], but in fully MHC-mismatched models, it has also been associated with a Th2 shift and prolonged graft survival [55].

CD155 (PVR), a nectin-like co-stimulatory molecule, balances immune activation through interaction with both activating (CD226/DNAM-1) and inhibitory (TIGIT, CD96) receptors on NK and T cells [56]. Naturally expressed at low levels on epithelial, endothelial, and antigen-presenting cells, CD155 is rapidly upregulated in response to stress and inflammation [57, 58], and localizes to interendothelial junctions, where it regulates the diapedesis of CD226^+^ leukocytes; blocking either CD155 or CD226 arrests monocytes and prevents their trans-endothelial migration [59]. In renal transplantation models, both CD155 and CD112 are constitutive, but their role in acute rejection has not been demonstrated [60].

Dual blockade of CD276 and CD155 almost completely prevented the increase in NK cytotoxicity observed under KIR–HLA mismatch, confirming these ligands as dominant checkpoints for missing-self recognition. In particular, CD155 inhibition curtailed early alloreactivity by dampening CD226-driven cytotoxicity and expanding FOXP3^+^Helios^+^ Tregs. It increased Treg frequency and CD25 expression, enhancing their IL-2-dependent suppressive ability, while also inducing PD-1/LAG-3 on effector T cells. These effects align with evidence that blocking CD226 signaling promotes Treg differentiation [42, 43, 45] which in the transplant setting is essential for both immune tolerance and tissue repair/engraftment [61].

These actions generate a more balanced immune milieu in which innate and effector T cell responses are attenuated, while Treg-mediated regulation could be reinforced. This creates a permissive niche that supports the engraftment and *in situ* maturation of iPSC-derived pancreatic tissues. Notably, these benefits are achieved without broad immunosuppression, thus preserving systemic immune competence and avoiding the detrimental effects that conventional drugs exert on lymphocyte and tolerogenic functions [62, 63].

*In vivo*, treatment with these targeted mAbs prolonged EP graft survival for up to 4 weeks, reduced innate immune infiltration, and enabled differentiation into functional insulin-producing β-like cells. The translational relevance of this approach is strengthened by the availability of fully human or humanized antibodies that have already been evaluated in oncology trials [36, 38], providing a feasible path toward clinical applications. Importantly, by modulating immunity during the period when iPSC-derived pancreatic tissues temporarily downregulate HLA class I [21, 64], our approach safeguards the grafts without requiring lifelong immunosuppression. Since transient HLA class I downregulation also occurs in cardiac [65], hepatic [66], and neural derivatives [67], this strategy may have broad applicability across stem cell-based transplantation.

Nonetheless, some limitations must be acknowledged. First, the observation window was limited to 28 days due to the onset of GVHD in PBMC-humanized mice, which prevented long-term follow-up. As a result, we cannot assess long-term graft survival, chronic rejection, allo-sensitization, or the development of donor-specific antibodies. Alternative humanized models lacking murine MHC class I and II, such as NSG MHC I/II double-knockout mice [68], could help address these limitations by lowering GVHD incidence and enabling longer-term evaluation of graft outcomes.

Second, while our data demonstrate that CD276/CD155 blockade promotes the sustained engraftment and *in vivo* maturation of iPSC-derived EPs over 4 weeks, this early “tolerance-like” phenotype should not be interpreted as definitive evidence of long-term immune tolerance. Secondary challenges, such as donor-matched skin grafting or long-term re-exposure to antigen, will be needed to assess if durable, antigen-specific tolerance has been achieved.

Additionally, we observed that a high dose of α-CD276 mAb accelerated GvHD onset, highlighting a narrow therapeutic window requiring optimization in future dose-ranging studies. Given that the immunomodulatory effects of CD276/CD155 blockade are transient, the primary risk may lie in increased susceptibility to infection during the peri-transplantation period. However, the duration and extent of immune modulation after treatment withdrawal, especially regarding antiviral and anti-tumor surveillance, remain unclear, and pathogen-challenge or tumorigenicity assays will be essential to understanding the long-term effects of the immunomodulation.

In conclusion, dual blockade of CD276 and CD155 emerges as a rational, early tolerance-promoting strategy that tempers innate immune checkpoints while enhancing regulatory T cell function. This immunomodulatory platform supports the engraftment and functional maturation of stem cell-derived pancreatic grafts and may serve as a foundational component of next-generation cell therapies. Future studies should assess whether combining this approach with adoptive Treg therapy or gene-edited hypoimmunogenic iPSC derivatives can achieve durable, drug-free tolerance in large animal models and, ultimately, in clinical settings.

## Data Availability

The original contributions presented in the study are included in the article/Supplementary Material, further inquiries can be directed to the corresponding authors.

## Supplementary Material

SUPPLEMENTARY TABLE S1 Immunogenetic features of the iPSC lines used in the study.

SUPPLEMENTARY TABLE S2 KIR genotyping of the NK cell donors.

SUPPLEMENTARY TABLE S3 KIR-HLA compatibility matrix between donor-derived NK cells and iPSC lines.

SUPPLEMENTARY FIGURE S1 HLA class I and differentiation marker expression dynamics during pancreatic commitment.

SUPPLEMENTARY FIGURE S2 Human immune cell engraftment in hIL-15 NOG mice infused with total PBMCs.

SUPPLEMENTARY FIGURE S3 Immune subset characterization in humanized hIL-15 NOG mice.
