# Supplementary material for: Blockade of CD155 and CD276 by Monoclonal Antibodies Fosters Immune Tolerance and Promotes Stable Engraftment of iPSC-Derived Islets in Allogeneic Humanized Mice
Source: Transpl Int. 2025 Dec 1;38:15433. doi: 10.3389/ti.2025.15433 (PMC12702790; doi:10.3389/ti.2025.15433)
Supplement: Supplementary file 1 [file Table1.docx]

**Table S1**

| **iPSC ID** | **HLA-A alleles** | **HLA-B alleles** | **HLA-C alleles** | **HLA-A allotypes** | **HLA-B epitopes** | **HLA-C allotypes** | **HLA-C exp. levels** |
| --- | --- | --- | --- | --- | --- | --- | --- |
| **AMF70.10** | 02:01 / 29:02 | 49:01 / 55:01 | 03:03 / 07:01 | - | **Bw4-I80^‡^/**Bw6 | **C1/C1** | Low/Low |
| **RP84.03** | 02:01 / 02:/05 | 35:01 / 50:01 | 04:01 / 06:02 | - | Bw6/Bw6 | **C2/C2** | Low/High |
| **AG89.04** | 02:01 / 11:01 | 35:01 / 51:01 | 04:01 / 15:02 | A11^†^ | Bw6/**Bw4-I80^‡^** | **C2/C2** | Low/High |
| **NL83.01** | 01:01 / 30:01 | 13:02 / 57:01 | 06:02 / 06:02 | - | **Bw4-T80/I80^‡^** | **C2/C2** | High/High |

^†^ Known to be recognized by 3DL2

^‡^ Isoleucine (I) or Threonine (T) in position 80 on aminoacidic chain

**Table S2**

| **NK ID** | **3DL3** | **2DL1** | **2DL2** | **2DL3** | **2DL5A/B** | **2DS1** | **2DS2** | **2DS3** | **2DS4-fl*** | **2DS5** | **3DL1** | **3DS1** | **3DL2** |
| --- | --- | --- | --- | --- | --- | --- | --- | --- | --- | --- | --- | --- | --- |
| **NK-26** | 2 | 2 | 0 | 2 | 1 | 0 | 0 | 0 | 1 | 1 | 2 | 0 | 2 |
| **NK-27** | 2 | 1 | 2 | 0 | 1 | 0 | 2 | 1 | 1 | 0 | 2 | 0 | 2 |
| **NK-28** | 2 | 2 | 0 | 2 | 0 | 0 | 0 | 0 | 0 | 0 | 1 | 1 | 2 |
| **NK-32** | 2 | 1 | 2 | 0 | 2 | 1 | 2 | 1 | 0 | 1 | 1 | 1 | 2 |
| **NK-33** | 2 | 1 | 2 | 0 | 2 | 1 | 2 | 1 | 0 | 1 | 1 | 1 | 2 |
| **NK-34** | 2 | 2 | 1 | 1 | 1 | 1 | 1 | 0 | 0 | 1 | 1 | 1 | 2 |
| **NK-48** | 2 | 1 | 0 | 2 | 3 | 2 | 0 | 1 | 0 | 2 | 0 | 2 | 2 |
| **NK-49** | 2 | 1 | 2 | 0 | 0 | 2 | 2 | 0 | 0 | 0 | 0 | 2 | 2 |

^*^ Full-length alleles preserving open reading frame only are reported.

**Table S3**

|  | **AMF70.10** | **RP84.03** | **AG89.04** | **NL83.01** |
| --- | --- | --- | --- | --- |
| **NK-26** | **Match (+2.0)** | **Mismatch (-1.1)** | **Match (+2.0)** | **Match (+4.0)** |
| **NK-27** | **Match (+1.0)** | **Match (+3.5)** | **Match (+6.6)** | **Match (+8.6)** |
| **NK-28** | **Mismatch (-2.0)** | **Neutral (+0.0)** | **Match (+0.2)** | **Neutral (+0.0)** |
| **NK-32** | **Match (+1.0)** | **Match (+0.6)** | **Match (+0.8)** | **Match (+0.6)** |
| **NK-33** | **Match (+1.0)** | **Match (+0.6)** | **Match (+0.8)** | **Match (+0.6)** |
| **NK-34** | **Neutral (+0.0)** | **Mismatch (-0.2)** | **Neutral (+0.0)** | **Mismatch (-0.2)** |
| **NK-48** | **Match (+3.0)** | **Mismatch (-8.0)** | **Mismatch (-10.8)** | **Mismatch (-13.0)** |
| **NK-49** | **Mismatch (-1.0)** | **Mismatch (-0.2)** | **Mismatch (-3.2)** | **Mismatch (-5.0)** |
