# Supplementary figures and images for: Blockade of CD155 and CD276 by Monoclonal Antibodies Fosters Immune Tolerance and Promotes Stable Engraftment of iPSC-Derived Islets in Allogeneic Humanized Mice
Source: Transpl Int. 2025 Dec 1;38:15433. doi: 10.3389/ti.2025.15433 (PMC12702790; doi:10.3389/ti.2025.15433)

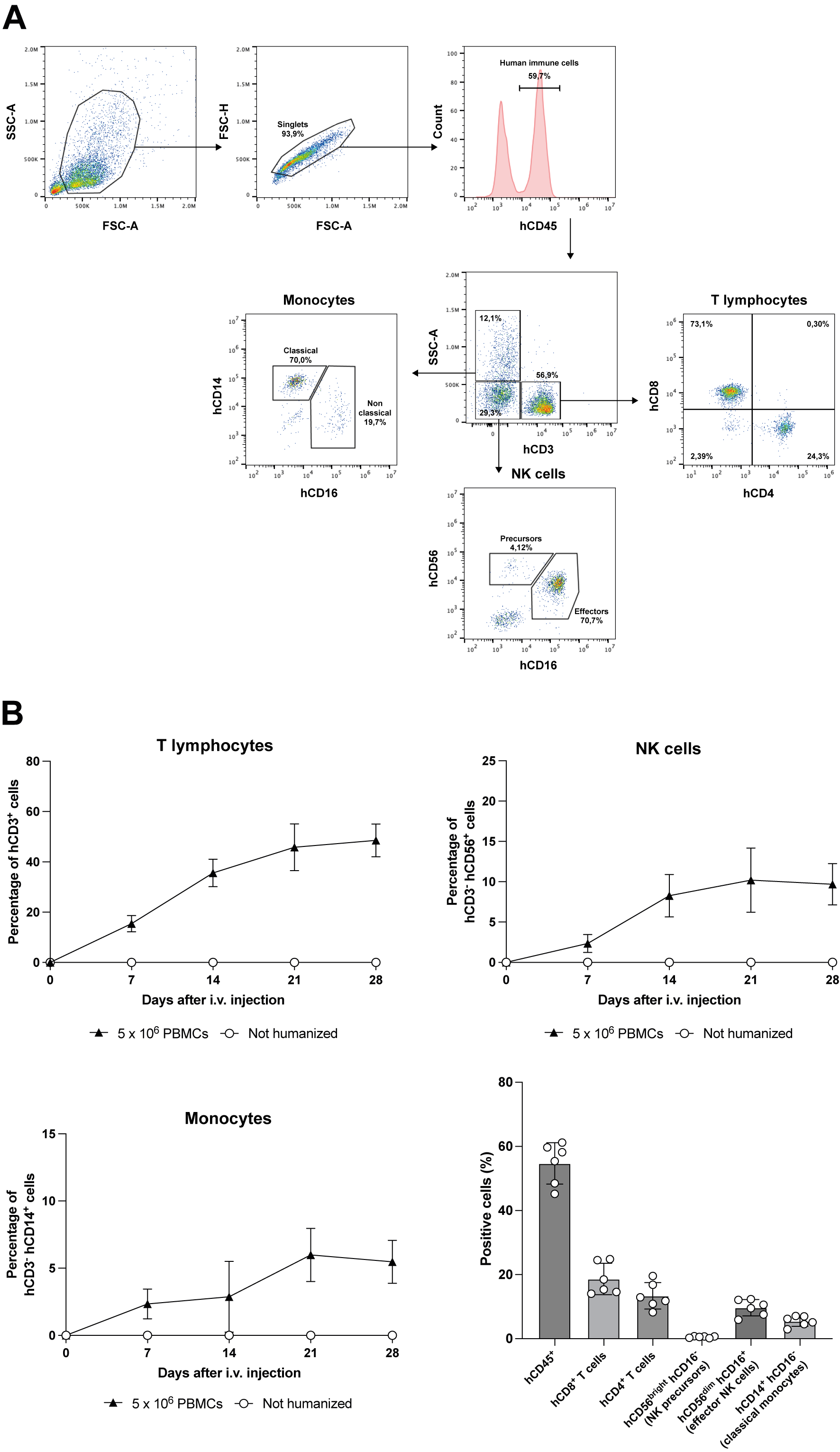

Supplement: Supplementary file 2 [file Image3.tif]

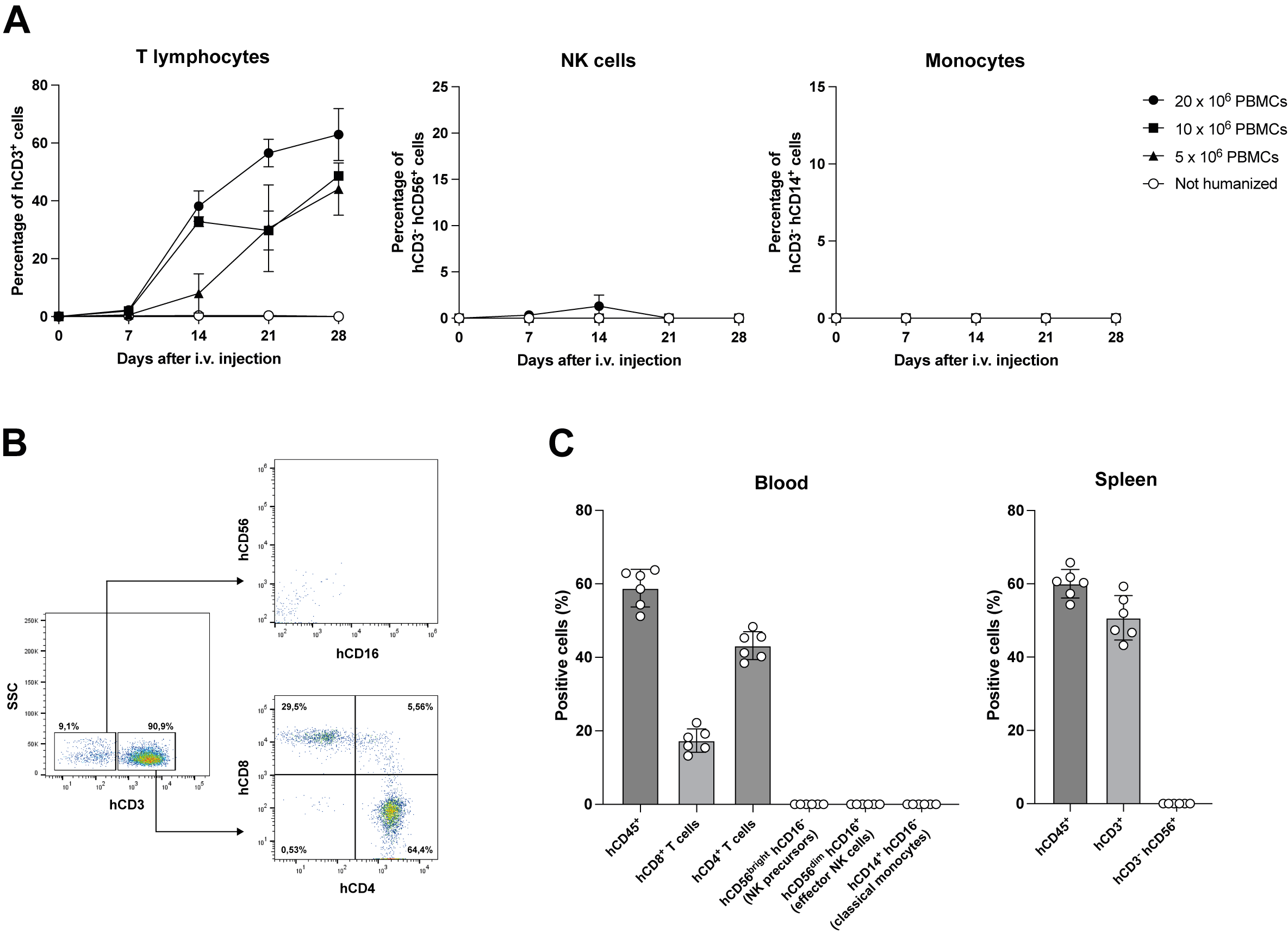

Supplement: Supplementary file 3 [file Image2.tif]

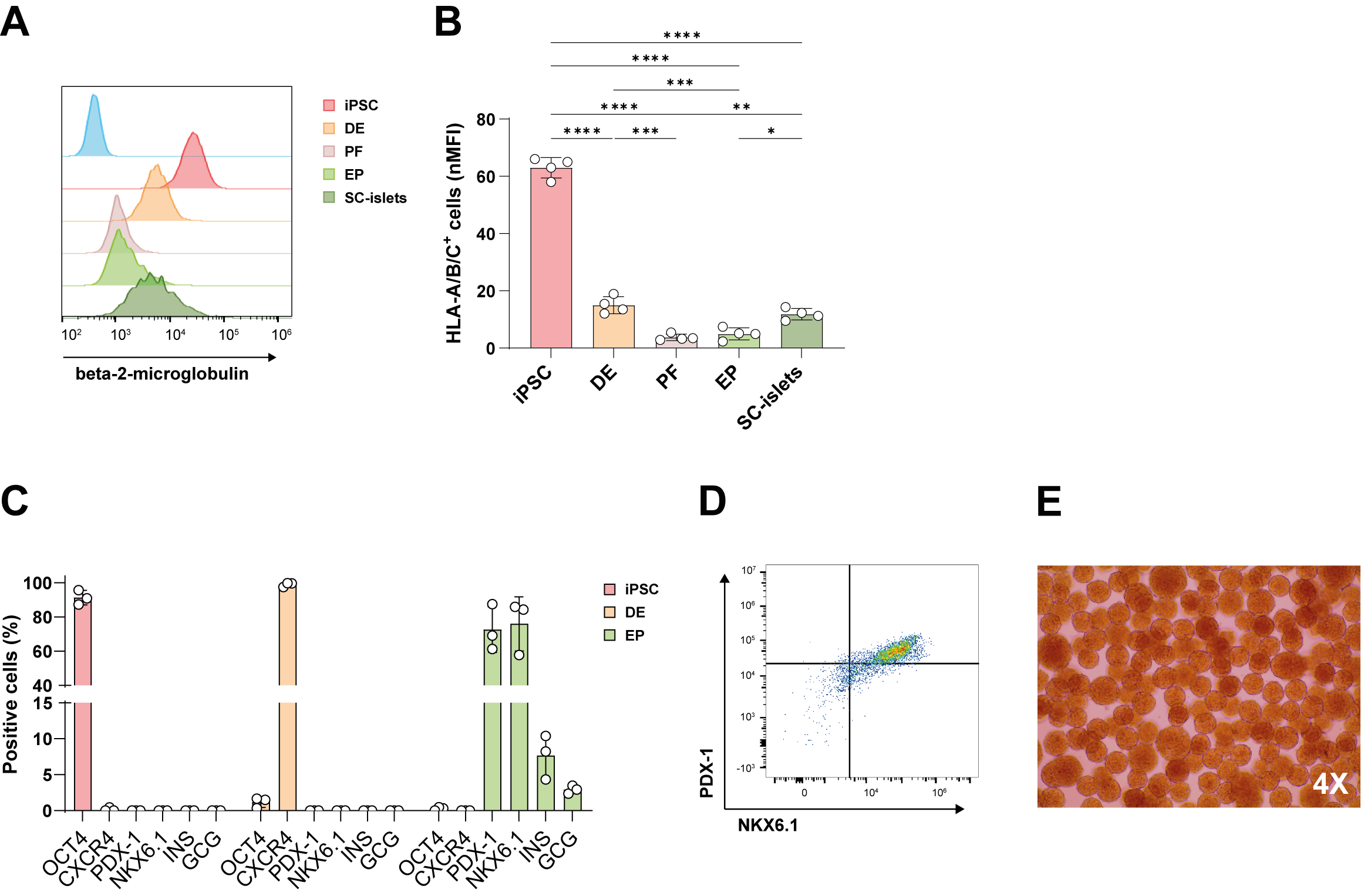

Supplement: Supplementary file 4 [file Image1.tif]
